# PreP+07: improvements of a user friendly tool to preprocess and analyse microarray data

**DOI:** 10.1186/1471-2105-10-16

**Published:** 2009-01-12

**Authors:** Victoria Martin-Requena, Antonio Muñoz-Merida, M Gonzalo Claros, Oswaldo Trelles

**Affiliations:** 1 Computer Architecture department, University of Málaga, Málaga, Spain; 2 Molecular Biology and Biochemistry department, University of Málaga, Málaga, Spain

## Abstract

**Background:**

Nowadays, microarray gene expression analysis is a widely used technology that scientists handle but whose final interpretation usually requires the participation of a specialist. The need for this participation is due to the requirement of some background in statistics that most users lack or have a very vague notion of. Moreover, programming skills could also be essential to analyse these data. An interactive, easy to use application seems therefore necessary to help researchers to extract full information from data and analyse them in a simple, powerful and confident way.

**Results:**

PreP+07 is a standalone Windows XP application that presents a friendly interface for spot filtration, inter- and intra-slide normalization, duplicate resolution, dye-swapping, error removal and statistical analyses. Additionally, it contains two unique implementation of the procedures – double scan and Supervised Lowess-, a complete set of graphical representations – MA plot, RG plot, QQ plot, PP plot, PN plot – and can deal with many data formats, such as tabulated text, GenePix GPR and ArrayPRO. PreP+07 performance has been compared with the equivalent functions in Bioconductor using a tomato chip with 13056 spots. The number of differentially expressed genes considering p-values coming from the PreP+07 and Bioconductor Limma packages were statistically identical when the data set was only normalized; however, a slight variability was appreciated when the data was both normalized and scaled.

**Conclusion:**

PreP+07 implementation provides a high degree of freedom in selecting and organizing a small set of widely used data processing protocols, and can handle many data formats. Its reliability has been proven so that a laboratory researcher can afford a statistical pre-processing of his/her microarray results and obtain a list of differentially expressed genes using PreP+07 without any programming skills. All of this gives support to scientists that have been using previous PreP releases since its first version in 2003.

## Background

Large scale gene expression monitoring technology is changing our view of the biological processes, including their dynamics. Hence, microarrays have emerged as the primary tool for studying the expression patterns of thousands of genes from a single experiment. As this technology matures, the ability to generate a large volume of data is accelerating; it is now perfectly normal to use tens or even hundreds of microarrays in a single study.

Microarray data are rich and complex but experimental biases, as well as variations introduced along the various steps in measuring gene expression levels, tend to make them unaffordable as is [1]. Therefore, data pre-processing is highly recommendable for reproducibility, reliability, compatibility and standardization of microarray analysis and results [2], even if it does not seem so necessary with Affymetrix chips [3]. Microarray data pre-processing, mainly normalization, is used to remove biases within each array by local regression. Many normalization methods often make the assumption that the majority of genes are not differentially regulated or that the number of up-regulated genes roughly equals the number of down-regulated genes. Although these assumptions are not applicable to every case, they do not seem to cause a serious effect on most microarray experiments. Alternatively, several methods have been proposed to normalize microarrays that do not fulfil the previous assumptions [4,5] for both Affymetrix GeneChip and two-colour array data. In any case, pre-processed data is usually more reliable in order to identify biologically meaningful patterns, since statistical tests to discover differentially expressed genes tend to depend on the experimental design.

An increasing number of academic and commercial solutions have been developed to tackle the pre-processing, each one with particular strengths and weaknesses. The most widely used and comprehensive packages currently belong to the open source software environment: Bioconductor for R [6], TM4 [7] and GEPAS [8].

Bioconductor is a collection of extensible open source libraries for R, whose main focus is to deliver a high quality infrastructure and end user tools for expression analysis. Object-oriented programming with well-defined classes is the basis for overcoming data complexity, and a command line interface is the preferred way to access libraries. This makes it very powerful but its use requires skills in statistics and programming capabilities. Data objects generated by R microarray processing packages can be saved in flat text being assimilated by the user, but the reconversion into the original object for further analysis is not always trivial. TM4 is a series of Java based tools that provide users with a well designed, easy to use interface. It consists of four major applications, as well as a MySQL database for maintaining experimental results, that are mainly focused on two-colour microarrays. In spite of its graphical interface, its use is not always intuitive and it also requires statistical skills to pipeline the pre-processing algorithms; it also presents certain computing inefficiency for intensive calculations. Unfortunately, only MeV is actually kept updated [9]. GEPAS is a nice and very used web based tool that allows the use of R packages without any programming skills. However, as most of the web based applications, it faces technological problems: poor interactive interfaces, not suitable for uploading and downloading huge amounts of data, lack on interactivity and data privacy problems, etc. Hence even if GEPAS deals with Affymetrix and two-colour experiments, its implementation presents some limitations.

Laboratory scientists are often challenged by large quantities of data produced by their microarray experiments, the statistics underlying the analysis of their own data, and the usability of applications that contain such statistical treatments. Pre-processing microarray data requires some background in statistics that most users lack or have a very vague notion of. This gap even includes the knowledge of which statistical approaches to use and the correct order in which statistical calculations have to be performed. In such a context, PreP has been in the front line of public software for two-colour microarray analysis [10], since it helps statistics-unskilled users to manage and analyze data effectively from their microarray experiments. It provides (a) an integrated gallery of techniques to deal with the many sources of measurement errors, including two new algorithms not available in any other tool; (b) an interactive user friendly interface for the visualization of data in an appropriate representation; (c) a standalone application for data privacy; and (d) highly customizable statistical tools to build up a simple error removal pipeline procedure. In spite of being designed as a tool for the analysis of two-colour chips, the data pre-processing of Affymetrix chips is possible through a slight initial preparation of data which consists of assigning one treatment to Cy3 channel and another treatment to the Cy5 channel; data can be thus processed and M and A values can be calculated. Automatic procedures will be soon added to PreP+07 to perform this data preparation. The improvements described here have turned PreP+07 into a user friendly environment that meets microarray pre-processing requirements for users that are not skilled in statistics or programming, but know how to perform a right experimental design concerning microarrays.

### Pre-processing methods available in PreP+07

#### Background correction and filtering

To enable comparison between arrays and experiments, data must be normalized and then replicates need to be resolved before differential expression analysis. Data treatment starts with background subtraction; this can be performed by PreP+07 or obtained from the microarray reading system. When data are supposed to be of high quality, subtraction can be enough; in any other case, background correction may need more artificial adjustments that are not available in Prep+07. Otherwise, PreP+07 has the option to start the normalization without background subtraction. Prep+07 also provides a data filtering tool to remove, for example, low quality spots, taking into account several criteria, such as foreground and background intensity, spot shape, saturation, etc.

#### Normalization

Typically, normalization is the first transformation applied to expression data. It aims to adjust the individual hybridization intensities to balance them appropriately so that meaningful biological comparisons can be made [11]. There are many approaches to normalizing expression levels, but the locally weighted linear regression (Lowess) normalization [12,13] has become the standard since it takes into account systematic biases and intensity specific artefacts that may appear in the data. PreP+07 implements both full parametric global and print-tip Lowess normalization procedure. Since normalised slides might not be comparable, scaling procedure is also provided for inter-slide normalization [13]. As a rule of thumb, no scaling must be performed unless box plots indicate that means of each slide are significantly different. However, some of the proposed methods are not supported by a model. These methods are called non-parametric and they offer, when properly used, a flexible approach to normalization.

#### Replication

Replication deals with the data merging from several repetitions of the same experiment and repeated spots in a single slide. Usually, errors cause data to be dissimilar from one repetition to another, but more knowledge about them is available as the number of replications grows. This information about error effects is collected by statistical procedures. Prep+07 can deal with biological and technical replicates by average (low replicate number), or by median calculations (when there are more than 16 values for each spot [11]). However, current proposals recommend using a noise (or error) model [14,15] and then extracting estimators [14], quality filters [16], thresholds [17], etc from it, to be taken into account in solving replications.

#### Double scan

This advanced correction method that improves data quality is uniquely implemented in PreP+07. Devices used for measuring intensities are neither perfect nor without limitations. Saturation and quantization, which compromise the high and low spot intensity reads respectively, appear in the scanned images, and are hard to be removed. The double scan method [18] combines two readings: a low intensity acquisition to avoid saturated spots and a high intensity second reading to avoid quantization, providing as a result a data set without saturation or quantization, so all slide spots become informative.

#### Supervised Lowess

Array based comparative genome hybridization (aCGH) is applied frequently to study the genomic content of closely related microorganisms, microbial taxonomy and species determination, as well as the presence of microbial pathogenicity factors. With aCGH a difference in signal arises, not only because of the absence or presence of genomic DNA, but also due to differences in sequence identity. This problem in particular plays an important role in bacterial aCGH experiments, since prokaryotes generally show lower genomic conservation than eukaryotes [19]. The Supervised Lowess (SL) normalization method only uses genes that are conserved (LHGs: likely homologous genes) in both samples hybridized for normalization. In a first step, the SL method performs Lowess normalization over the LHG subset of genes, computing the initial log ratios (i.e. Ri (i = 1...N)), followed by Lowess normalization, generating a set of corrected ratios Rc i (i = 1...n, n < N) and correction factors for the subset of conserved genes used: αi = Rc i - Ri. Subsequently, the Lowess correction factors belonging to the subset of conserved genes (αi) are extrapolated to determine the correction factors βj (j = n+1...N) for the remaining genes. The correction factors are then used to adjust the log ratios of the remaining genes. The spot set used for SL can be selected by hand or using the filtering capabilities of Prep+07.

#### Differential expression

Finally, differential analysis serves to identify outlier spots (differentially expressed ones) whose outlying behaviour is not due to experimental error but biological expression. The differential expression based on a fixed fold change cut-off has been identified as insufficient. Therefore, methods involving calculation of the mean and standard deviations [16,20] of the spot distribution of log2(ratio) values, and also defining a global fold change difference and confidence [21], equivalent to a z-test, have been included for a preliminary analysis.

#### A typical protocol

**Figure 1:**
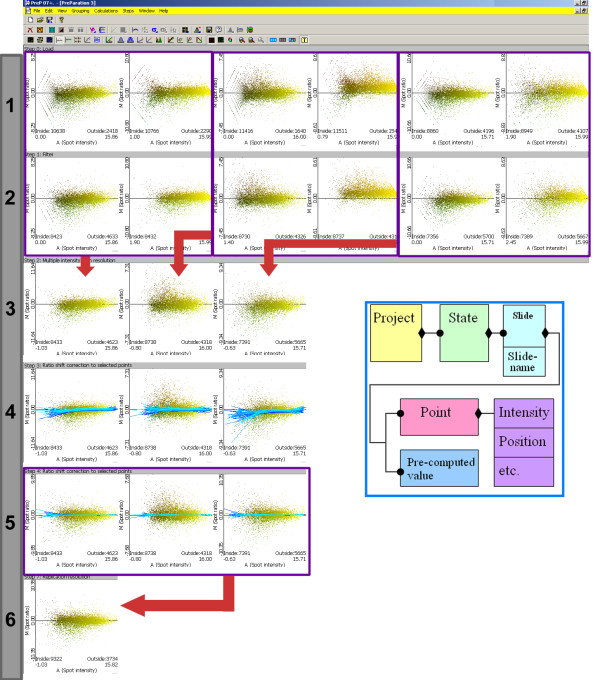
**Typical steps in a complete analysis of gene expression**. (by row): (1) Filtering empty spots; (2) double scan resolution; (3) Lowess estimation of parameters; (4) applying the Lowess estimation; and (5) Replicates resolution. Inside the box: object diagram of a PreP+07 project, where diamonds represent "is composed of" and circles represent "one or more" (6) final result.

A typical normalization procedure (Figure 1) using PreP+07 starts loading data to which column-functionality is assigned (pre-defined format files can be used to automate this step). Frequently, a row filtering step is needed to remove low quality and empty spots. Several options are available for filtering, such as spot quality, signal presence/absence, fold change, etc. When applicable, a 2 scan resolution can be performed to extend dynamic range of intensity values. Data normality can be visually evaluated using normality graphs and then data normalization can be chosen to correct the deviation (lowess or supervised lowess). Statistical tests can be used to identify differentially expressed genes. Finally, results can be saved in several formats for further processing.

## Implementation

The methods described above have been implemented in PreP+07 [see Additional File 1 for the description of the main sources of experimental errors and the solutions provided by PreP+07]. Among them: merging intensities by double scan method*; adjusting by Lowess or Supervised Lowess regression*; scaling for inter-slide normalization; filtering* and thresholds; random error removal by replicates resolution; global and local statistical descriptors such as z-test, t-test, p-values; etc. ("*": new in PreP+07; see Table 1). The general setup of PreP+07 offers a certain degree of freedom when running pre-processing methods. A remarkable feature in this version are the Normality Plots (Quantile-Quantile, Probability-Probability, Probability-Normal), that help the user to visualize the normality of the data. When the observed data nearly follows the expected distribution, a diagonal line will be drawn and in this way outliers will be easily observed on these graphs as points both ends of the diagonal.

**Table 1:** Methods available in PreP+07 vs PreP 2003 version

**PreP Methods**	**PreP 03**	**PreP+ 07**
Background correction	Y	Y
Logratio conversion	Y	Y
Block division	Y	Y
Filtering	N	Y
Double Scan	Y	Improved
Lowess per block	Y	Y
Supervised Lowess	N	Y
Scaling – Standard Deviation/Median Absolute Deviation – Intraslide/InterSlide	Y	Improved
Replication	Y	Improved
Dye Swap	Y	Y
Stat Test – Local/Global – Ztest Ttest	N	Y
**Graphics**		
Threshold lines	Y	Y
Slide View	Y	Y
Coherent Slide View	Y	Y
Quality Slide View	Y	Y
MA Graph	Y	Y
MA Quality Graph	N	Y
MA per blocks	N	Y
RG Graph	Y	Y
Box graph	Y	Y
Normality graphs (QQ/PN/PP)	N	Y
Density Graph	Y	Y
Density Graph per Block	Y	Y
DoubleScan graphs	Y	Y
Logratio histogram	Y	Y
Replication graphs	Y	Y
**Others**		
Zoom	Y	Y
Online help	N	Y
Open/Save project	Y	Y
Save expression Matrix	Y	Improved
Automatic load of genepix, imagene files	N	Y
Loading formats automatically	N	Y
Delete last step	Y	Y
Delete all steps except last	N	Y
Toolbar redesigned, related buttons consecutive	N	Y
Slide Alias when you load it	N	Y
Apply the same structure with a checkbox button to all loaded slides	N	Y
Tooltip activation button	N	Y

### PreP+07 Input/Output

Special attention has been paid to improve input-output functionality in PreP+07. Input and output files in PreP+07 are tab-delimited text files, which can be readily imported, for instance into Microsoft Excel. In addition, PreP+07 manages its own data format (engene compatible [22]), and compatibility with Genepix (*.grp), ArrayPRO and text-tabulated output files are also provided. Additionaly to data loading, the meaning of each column must be specified (column functionality setting). Manual or file configuration can be used for this purpose, including the description (sectors, print-tip groups or grids) of slide structure (*.CEL files from AffymetrixTM platform are accepted as well).

PreP+07 also supplies a broad range of alternative output formats, from simply a text tabulated table to gene expression matrixes including statistical characterization. An important and useful feature is the ability to store intermediate results as a PreP project that can later be recovered for further processing.

### Double Scan

**Figure 2:**
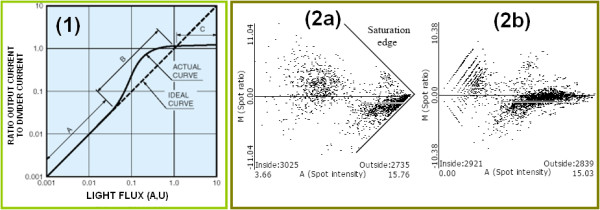
**Double-Scan procedure**. In (1) the transfer function of the photo detectors used in the scanners is depicted. At high intensities the relationship between the incident light level and the output current begins to deviate from the ideal intensity in an effect called saturation that is typically drawn in an arrow shape (see 2a). On the other hand, quantization occurs when digitizing. All the unlimited physical values have to be encoded by a reduced set of discrete values, producing the same rate for a range of different values [21]. This effect can be observed in (2b) as a set of parallel lines. 2-Scan strategy [16] is based on the rather simple idea of producing two images with different calibrations, from which a mathematical model produces a coherent but extended range of values.

Double scan implementation is based on a robust mathematical model described in [18] and requires the identification of the saturation model (clipper or gamma). Adjustments can be visualized with the 'intensity-intensity' plot. It shows the spots according to the intensity measured in a low sensitivity scan and a high sensitivity scan for both the green and red channels. This plot can also be used for the comparison of two replicated slides or scans. If the replication is properly done, the spots must show a linear relation; otherwise, when the scans have different calibrations, the data will follow a non linear curve due to saturation or calibration effects (see Figure 2).

### Supervised Lowess

Supervised Lowess (SL) can be advantageously used when data follow a non normal distribution due to differences in gene sequence identity, as demonstrated in [19], suggesting that it is appropriate for any microbial aCGH comparison. In any case, SL assesses a normalizing estimate using a subset of genes (sharing strong sequence similarity) and then uses this estimation to remove the error in the rest of genes. This procedure has been successfully applied to spiked-in dual dye DNA microarray data.

### Visualization tools

There are many ways to represent microarray data (MA plot, QQ plot, RG plot, etc.), many of which have been implemented in PreP+07 (see Table 2) in order to help in data interpretation as well as to detect any kind of systematic error in the dataset. In a first instance it provides a synthetic image built from the image processing software output that gives a general overview of the data quality and can be compared to the original scanner images, not only to validate the reading of data but to aid in the discovery of any unnoticed artefact or incoherent values due to noise of bad background estimation. The MA plot [12], which represents the intensity (A) against log2(ratio) of expression (M) is nowadays an indispensable tool in microarray representations. It can help locate outliers, detect any kind of quantization at low intensity or saturation at high intensities. In PreP+07 the Lowess normalization curves are drawn on these plots in order to assess the degree of adjustment that the normalization will introduce.

**Table 2:** Visualization tools available in PreP+07

**Name**	**Method**	**Use**
Slide view	A synthetic reproduction of the scanned image from the available data.	Comparison with the scanned image, identifying single spots, splitting the slide in blocks and manual testing.

Slide view of coherent spots	A synthetic reproduction of the scanned image only for coherent data.	Evaluation of the quality of the slide and poorly scanned zones (negative or null values are not shown).

Slide view with quality	Uses the blue channel for displaying the quality of the measure.	Combined with algorithms that provide a quality value for each spot.

AM and RG Graphs	(AM) Logarithmic plot of ratio versus intensity; or (RG) log. of red versus green channel	AM displays the dependencies of the ratio on the intensity (ratio correction and filtering); in the (RG) case the two color channels are emphasizing separately.

Box Graph	Box graph of each block of the slide.	Classical statistical graph for detecting outliers and comparing the distribution of diverse data sets (useful tool for detecting contrast variations inter- or intra-slide).

Density Graph and Density Graph per block	This graph estimates the density of ratios (per block).	Preliminary test on the distribution of the ratios. The expected density graph is a normal distribution (per block, helps detecting spatial errors).

Intensity-Intensity Graph	A scatter plot showing the intensity values of one scan acquisition versus the same values of another scan acquisition.	This is a first step for comparing two slides. The data should be near the diagonal if the slides are good replicates of each other.

Dispersion, Deviation and Correlation of Replicates	The intensity values of the individual spots versus the mean of all the spots from the same replication group.	Quality estimation of the replication. For dispersion graph, the data points should be along the diagonal, and the more noise, the more blurred they will be. If the deviation is high the quality will decrease

Normality of Replications	Applies the inverse of the normal distribution function to the distribution function of each replication group.	One typical assumption is that the noise is normally distributed. This graph will test that hypothesis. If the data points lie along the diagonal, the noise is normal.

Probability Normal Plots (PP/QQ/PN)	Plots to compare expected normal distribution values against observed values	QQ compares z-scores, PP p-values and PN compares pvalues vs logratios

Replicated data can be visualized, and the quality estimated, by Dispersion, Deviation and Correlation diagrams that expose these statistical values and their dependence on the average of the replicated spot intensities. For the dispersion graph, the data points should be located along the diagonal, and the more noise, the more blurred they will appear, in other words, a lot of spread spots suggests low data quality.

To assess the replication normality, QQ plot compares quantiles of the expected normal distribution with quantiles of the observed data distribution (similar to QQ, the PP shown p-values and the PN draws p-values versus log ratios). These plots are drawn for every step in the project stack.

**Figure 3:**
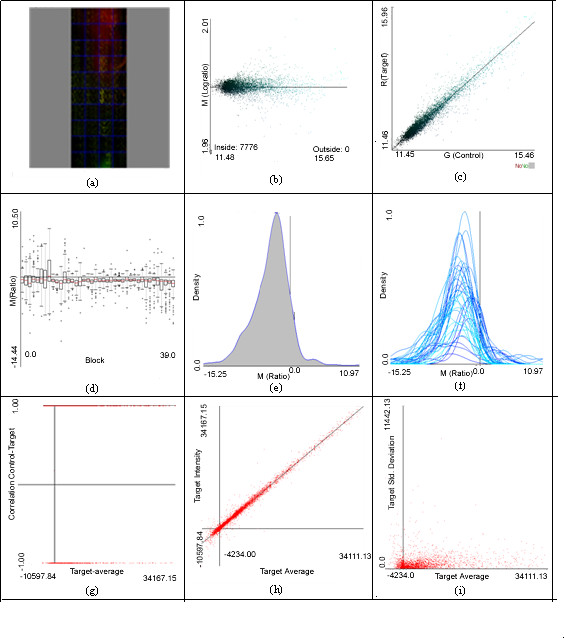
**Some PreP+07 views**. a) slide view, (slide view of coherent data, slide view with quality also available) b) MA plot, c) RG plot, d) box graph, e) density distribution of ratios, f) density distribution of ratios within each sector, g) correlation of replicated spots vs. their average h) normality of replications i) deviation of replicated spots vs. their average.

To emphasize PreP+07's user friendliness most of the graphs have interactive visualizations: tooltips with the data associated to each spot, colours that help the user differentiate genes with differential expression, etc. Clearly, PreP+07 outperforms existing implementations of gene expression graphical representations (see Figure 3).

### Local Normalization

PreP+07 implements a local deviation procedure to get a preliminary set of differential expressed genes for this issue with three different estimators: (a) windowed local deviation that takes a fraction of spots near the spot whose deviation is to be found, and then it uses those local spots for the estimation; (b) Lowess absolute deviation, that uses a Lowess curve, given a fraction and a number of steps, for absolute deviation fitting, and (c) Lowess standard deviation, similar to (b) but for standard deviation fitting [12,13] Negative ratios can be managed as symmetric, forcing the deviation to be the same for positive and negative values, or as asymmetric, to allow different deviations for positive and negative values.

### Architecture implementation

PreP+07 is implemented in Visual C++ for the MS-Windows XP OS. It is designed in an object oriented way for robustness and scalability. The code is intended to ease the use of the application. An important goal was making the user interface friendly. This is achieved by extensive visual information, using the operative system's GUI libraries and a high degree of interactivity. The installation of PreP+07 is extremely simple, just downloading the software from the Web site and launching it. A comprehensive user-friendly manual is also available, giving more details about the methods used, and a pertinent guided tour allows a step by step discovery of the software.

#### A PreP+07 project

Conceptually speaking, a PreP+07 project is a collection of states. Each state is the result of applying a given process over the previous state. The different PreP+07 states are stored in a stack, meaning states are pushed into the stack and only the last state can be removed (popped-up) from the top of the stack. The last state is the current state, this is to say, the state over which the procedures are applied (the rest of states conform the "history"). Each state is self-contained so that it holds all the necessary information to produce a new state (this allows using a test-error approach to obtain the best results).

Each state consists of a collection of slides. The slides represent and contain the information obtained by the scan of a given DNA chip. The slide has an associated name and, when necessary, a set of pre-computed values to be used in a new step. In general, the slide name resumes the experimental conditions. Finally, a slide is a collection of points (spots). Each spot has a set of values that correspond to light intensities, position in the chip, labels, etc. (see Figure 1).

The first state is produced by a special step named the "load step". In this step the slide files are loaded and identified to translate the original data tags into PreP+07 understandable tags. Options available for the "load step" are particular to this stage (and different for the next "normal" steps). Some of the different procedures implemented in PreP+07 can be applied in any context (such as the normalization; adjusting and ratio scaling) while others require specific conditions (e.g. gene replication).

## Results and Discussion

Since Bioconductor packages are considered a standard in microarray analysis, PreP+07 results were compared with it. The comparison rationale has been to obtain normalized log-ratios by applying R and PreP+07 procedures, then use these log-ratios to perform a two-class t-test and detect the differential expressed genes in both datasets using the Multi Expression Viewer (MeV) program from the TM4 [7].

A complete set of experimental data obtained in the framework of ESPSOL Spanish project [23] with Solanum lycopersicum has been used to obtain a set of differentially expressed genes, following the typical protocol described previously. The set was composed of 6 tomato microarrays hybridized to samples representing two different conditions, (three biological replicates for each one called A1, A2, A3, and B1, B2, B3). To keep data confidentially, random Gene IDs were assigned for the tomato sequences. The experimental design includes a dye-swap and images were obtained with the GenePix technology. These chips are organized in 4 × 12 blocks (row major) and each block contains 16 rows and 17 columns (13056 spots), including 896 empty and intra-slide replicates for some tomato ESTs and negative controls, identified by the same ID. In particular 140 different spots contain 14 different negative controls (belonging to different species) and 174 spots contain replicates for 16 ESTs. So finally, 12020 spots correspond to tomato sequences in the chip [see Additional File 1]. All scan acquisitions were performed at normal intensity (PMT GAIN = 730V × 610V) with a minimal number of saturated signals (less than 0,55% in all cases).

**Figure 4:**
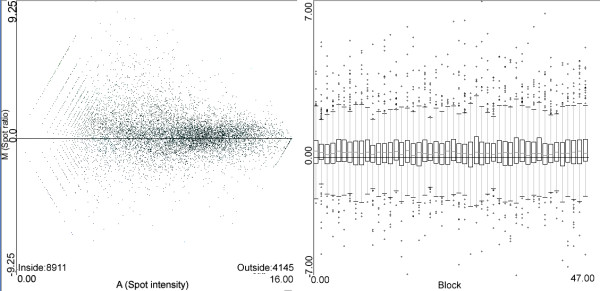
**AM and boxplot graphs on one of the initial dataset**. Quantized low quality values can be observed in the low intensity zone of AM graph suggesting the need for a filtering procedure, and the nice shape in the boxplot (on the right hand side) suggests that scaling procedure is unnecessary.

Once empty spots were removed (Figure 4), data were pre-processed to obtain log-ratios following two protocols in order to allow for software comparison. They are FL (filtering low quality and normalizing with Lowess) and FLS (FL plus scaling). Both protocols were applied to microarray data using PreP+07 and using the Bioconductor package Limma.

**Figure 5:**
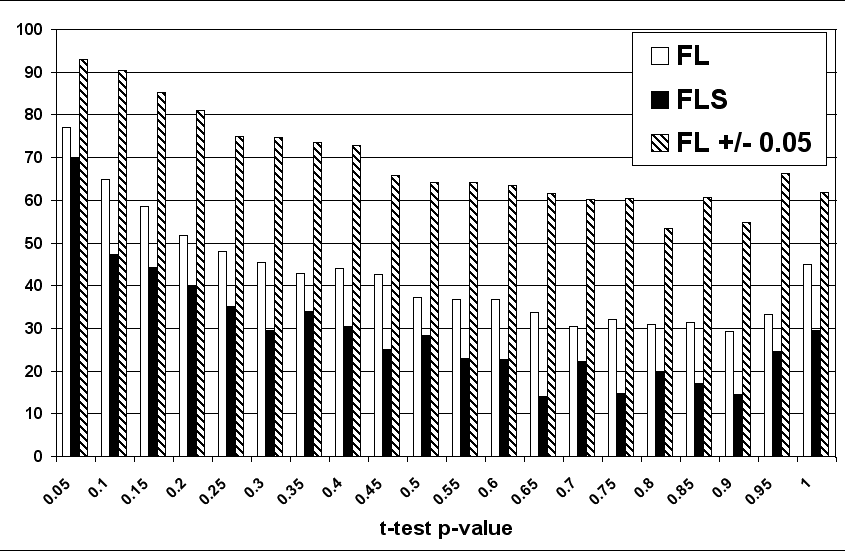
**Percentage of predicted genes by Limma in the same p-value range of PreP+07 predictions**. White bars belong to protocol 1 (FL), black bars correspond to protocol 2 (FLS) and slashed bars belong to protocol FL with neighbouring (a range of ± 0.05). Note the high coverage value (> 90%) for the most significant genes (p-value < 0.1) and that fact that major differences are produced in the low quality expression levels. The general coverage is approximately 70%.

Filtering step is designed to remove low quality spots [see Additional File 1] and resulted in 5966 filtered spots with detectable signal in all 6 samples. The log-ratios produced by Limma and PreP+07 were used as input for MeV to perform a two conditions t-test. This test provides a p-value that ranks genes whose mean expression level in group A is significantly different from the mean expression level in group B. The complete set of genes was classified in 0.05 wide ranges following their p-values. The number of differentially expressed genes for p-values coming from PreP+07 and Limma were nearly coincident (Figure 5) considering ± 0.05 fluctuations. It should be noted that the FLS protocol produces a smaller correspondence, which supports the idea that scaling introduces more noise than benefit.

In order to learn more about the correlation between results, genes coming from PreP+07 pre-processing with a t-test p-value < 0.01 were selected and sorted according to their p-value and then compared with their counterparts as calculated with Limma (see Table 3). This procedure allows knowing what differences would be obtained if PreP+07 or Limma were used for gene selection. The main result differences correspond to genes 10247 and 2213, so they were analyzed in detail from their log-ratios to the final p-values to better understand these differences (Table 4). As can be seen on the table, differences in log-ratios in the t-test p-value are produced by slight differences in the means and their deviation values, which result in higher differences between groups and low intra-group variability.

**Table 3:** Differentially expressed genes obtained with the FL protocol using PreP+07 contrasting their rank-position against Limma ranking.

[1]** ID**	[2] **P+07 pvalue**	[3] **P+07 rank**	[4] **R pvalue**	[5] **R rank**	[6] **pvalue difference**
2108	4.92E-04	1	5.54E-04	1	6.20E-05
8057	0.00277	2	8.91E-04	2	1.88E-03
269	0.00318	3	3.259E-03	3	7.75E-05
3677	0.00376	4	0.00845	9	0.00469
8708	0.00408	5	0.00384	6	0.00024
6174	0.00661	6	0.01569	25	0.00908
11844	0.00665	7	0.00378	5	0.00287
10247	0.00738	8	0.03051	61	0.02312
9724	0.00831	9	0.01275	19	0.00444
1783	0.00907	10	0.00980	12	0.00072
10585	0.00952	11	0.01106	14	0.00153
2213	0.00997	12	0.02626	46	0.01628

Columns correspond to: [1] gene ID; [2] and [4] t-test p-value for data processed by PreP (in increasing order) and Limma; [3] and [5] position in the list of significant genes in PreP+07 and Limma; [6] differences in p-value: | [2] – [4] |. T-test p-values were obtained using MeV from TM4 package.

**Table 4:** Detailed information of spots 10247 and 2213.

**Gene**	**Logratio**	**A1**	**A2**	**A3**	**B1**	**B2**	**B3**	**Mean A**	**Mean B**	**Dev A**	**Dev B**
10247	PreP+07	0,1234	0,6886	0,1747	-1,0292	-0,8200	-1,1466	0,3289	-0,9986	0,3126	0,1654
	R	0,0879	0,7503	0,1842	-0,9922	-0,7512	-1,0886	0,3408	-0,9440	0,3579	0,1738
	Differences	**0,0356**	**0,0617**	**0,0095**	**0,0370**	**0,0688**	**0,0580**				

2213	PreP+07	-1,1997	-0,7258	-0,5227	0,3122	0,6829	0,5608	-0,8161	0,5186	0,3474	0,1889
	R	-1,1975	-0,6150	-0,5268	0,4582	0,7352	0,5584	-0,7798	0,5839	0,3644	0,1403
	Differences	**0,0023**	**0,1108**	**0,0041**	**0,1460**	**0,0522**	**0,0025**				

For these genes, log-ratios (both from Prep+07 and R and their absolute value difference) for the 6 analyzed chips are shown including mean and standard deviation for conditions A and B, from which the p-value was estimated.

An additional experiment with no proprietary dataset has been performed using a public dataset from GEO (accession GPL7275). Samples belong to NK cells of C57BL/6 mice either mock-infected or infected with P. chabaudi with ID codes from GSM319497 to GSM319502 (3 samples per condition) (see a complete description in ). The object is to identify differentially expressed genes by the infection with P. chabaudi. The protocol used for the analysis was the same but empty or low quality spots were not removed. This data set also shows a significant agreement between R and PreP results [see Additional File 1].

## Conclusion

Pre-processing is a necessary step when preparing gene expression data for analysis since raw data carry instrumental and operator errors. Moreover, these biases are not constant across experiments, rendering the data inconsistent. Furthermore, the preprocessing methods should keep real differential values (over- or under-expressed genes) still identifiable, and this must be achieved by using outlier detection and robust statistical methods.

PreP+07 is an attempt to reduce the barriers between scientists that hybridize microarrays and statisticians that analyze microarray results in depth. In other words, PreP+07 enables scientists to prepare their data and conducts a basic analysis of differential expression which is ready for closer and more specialised inspection. Hence, PreP+07 has been designed a standalone interactive graphical suite to integrate widely used pre-processing methods for gene expression data that aims to minimize sources of systematic and random variation in the acquired data, other than those directly related to differential expression. PreP+07 includes a variety of analytical tools for reducing dependencies of intensity and, when available, allowing the resolution of replicated data sets. In some cases these can be applied in any context (such as the normalization, adjusting and ratio scaling). In other cases, some specific conditions have to be met (e.g. gene replication). Once the error has been minimized, PreP+07 allows extracting the individual control, target signals and their ratio since most of the techniques available on PreP+07 are based on robust statistical procedures, thus being respectful to outliers and differentiated values.

Statistical microarray analyses (e.g. Limma/Bioconductor) require a collection of biological and technical replicates in order to obtain information about what genes are differentially expressed. In addition to this, PreP+07 also provides the opportunity of analysing differentially expressed genes slide-by-slide by means of a t-test or z-scores statistics. Slide-by-slide analysis can be very helpful for researchers unskilled in statistical methods that want to obtain an overview of their results. These advantages are strengthened by the interactive interface of PreP+07, which allows the identification of values and quality of every spot on the slide in each plot. The available plot set enables data visualization using different criteria to assess data reliability.

Among the multiple advantages of using PreP+07, the most remarkable characteristics are (a) the visualizations tools are completely interactive, with optional tooltips for each coloured spot in the graph to display complete information aimed to identify outliers spots and obtain their information visually (including tracking information about the number of coherent/incoherent or filtered spots); (b) new and unique methods such as Supervised Lowess and double scan regression; (c) intuitive and powerful replication resolution that allows users to combine inter- and intra-slide replicates; (d) comparable results with most used related software allowing non-bioinformaticians to do the same pre-processing procedures using a graphical and intuitive interface, ensuring data privacy and high quality images; and (e) data results and inputs are interchangeable between programs (i.e. R output can be loaded into PreP to realize different analysis and vice versa).

The learning curve in PreP+07 can be expected to be smoother than the learning curve in R Bioconductor, with PreP+07 the biologist does not need to have prior knowledge about scripting and simple steps such as loading the data and applying filtering or lowess could be done intuitively the first time the user runs the program.

PreP+07 is intended for preliminary microarray analysis for users unskilled in statistical microarray treatments or without scripting languages' capabilities. This is why it is an integrated application that contains only well-known and widely used methods (not all available methods or applicable methods) such as print-tip-lowess, lowess or scale. The idea is not to open a wide range of opportunities, but to offer a small collection of reliable workflows with the necessary options to reach normalised data and even a set of differentially expressed genes.

PreP+07 has been exhaustively tested in various research projects, like aCGH with spiked-in dual dye [19], Express Fingerprints [24], Gene expression pattern and protein profile in pigs infected by circovirus [25] and ESP-SOL Project [23].

## Availability and requirements

▪ Project name: PreP+07.

▪ Project home page: 

▪ Operating system(s): Windows XP.

▪ Programming language: Visual C++.

▪ Other requirements: none.

▪ License: free software.

▪ Any restriction to use by non-academics: none.

## Authors' contributions

VMR designed and programmed the new methods and improvements of Windows PreP+07 program. AMM carried out the transcriptomic analysis and exhaustibly tested the application. MGC tested the application and helped with the manuscript. OTS conceived of the study, participated in its design and coordination and helped to draft the manuscript. All authors have read, participated in, and approved the final manuscript.

## Supplementary Material

Additional file 1 **Supplementary material**. This document contains the different sources of error on microarray data analysis, a full table with the existing applications in gene expression data analysis, a typical PreP+07's protocol of use, an alternative experiment comparing PreP+07 and R, Technical details of PreP+07 code and the manuscript's figures in full resolution. Click here for file
