# Supplementary material for: PreP+07: improvements of a user friendly tool to preprocess and analyse microarray data
Source: BMC Bioinformatics. 2009 Jan 12;10:16. doi: 10.1186/1471-2105-10-16 (PMC2657788; doi:10.1186/1471-2105-10-16)
Supplement: Additional file 1 — Supplementary material. This document contains the different sources of error on microarray data analysis, a full table with the existing applications in gene expression data analysis, a typical PreP+07's protocol of use, an alternative experiment comparing PreP+07 and R, Technical details of PreP+07 code and the manuscript's figures in full resolution. [file 1471-2105-10-16-S1.pdf]

Supplementary Material

PreP+07: improvements of a user friendly tool to pre-process and analyse microarray data

Victoria Martín-Requena[1], Antonio Muñoz-Mérida[1,] M. Gonzalo Claros[2] and Oswaldo Trelles[1,*]

[(1)] Computer Architecture department, University of Malaga Spain.

[(2)] Molecular biology and biochemistry department; University of Malaga, Spain

Contents

1 Sources of error, visualization and correction procedures in microarray experiments
1.1 Artefacts
1.2 Ratio shifting
1.3 Ratio stretching
1.4 Spatial effects
1.5 Data acquisition
1.6 Random errors
1.7 Other visualizations
2 Available software packages for gene-expression data pre-processing
3 Processing Protocol
3.1 Steps in a typical analysis using PreP+07
3.2 Filtering Flagged spots
3.3 Graphical representation of initial data quality for the slides used in the tomato experiment
3.4 Notes about replication
3.5 Details about using R
4 Results and figures from Mus musculus experiment
5 R use analysis
6 Technical details of Prep+07 code
7 Basic Classes diagram used in PreP+07
8 Manuscript's images in full resolution
9 Supplementary material’s figures

1. SOURCES OF ERROR, VISUALIZATION AND CORRECTION PROCEDURES IN MICROARRAY EXPERIMENTS

1.1.- Artefacts

Artefacts are very strong errors that occur at very specific locations. They are usually caused by small accidents such as blots or fibers on the slide. Most image processing software packages detect these artefacts and mark the corresponding spot for the user to check them. However, some invalid spots may remain unnoticed. PreP+07 allows a visual comparison of the original image and a synthetic image built from the image processing software output. This comparison is helpful for determining artifacts that have gone unnoticed. Also, this synthetic image, called 'slide view', shows spatial dependencies and allows the user to divide the slide in sectors of homogeneous characteristics. There is a mode of the slide view that only shows the spots whose intensity values are coherent, i.e. non negative values. The incoherent values are produced by noise or bad background estimation. The main aim of this 'slide view of coherent spots' is to aids in assessing the quality of the slide, by detecting the zones of the slide that have been poorly scanned.

1.2.- Ratio shifting

The results of the microarray technology lie in the ratio of the target and control measures. If a kind of error modifies this ratio, shifting it from its real value, it must be corrected or compensated before proceeding to analysis. Many of the causes for this shift are systematic and can be predicted fairly well. Different dye incorporation and fluorescence efficiencies, scanning sensitivity or laser power variation for different colours and non-linear transfer functions in photodetectors are behind these sources of error. A common way to evidence any dependency of the ratio on the spot intensity is the MA plot (Dudoit et al. 2001) which is provided by PreP+07. There must not be any dependency and both ratio correction and filtering will remove it. PreP+07 provides a ratio correction and filtering procedures.

1.3.- Ratio stretching

Ratio can be not only shifted, but also stretched irregularly over the slide and between slides. Causes of these contrast variations are nonlinearities, environmental conditions when measuring, scanner focusing, calibration and sensitivity. These errors appear when both channels are amplified in a non-proportional way and they can be compensated by a scaling procedure. The box graph is a very used statistical plot that depicts the contrast variations. It shows the median and adjacent quartiles in a box and an estimation of the spread of the data. Single data points outside the estimated spread are individually plotted as small circles above or beneath them. This schema is repeated in every slide sector for the user to be able to compare the distribution of the data between these sectors. If the contrast were uniform, all the boxes in the box graph would have the same height. PreP+07 provides both a scaling procedure and the box graph diagram.

1.4.- Spatial effects

Neither the slide where the spot are placed is not a perfect and balanced surface nor the solution used for hybridisation spread it uniformly. The printing needles used for spotting could be dissimilar and, even, in the same printing tip, the volume of liquid changes from time to time. When scanning, the lighting may be irregular or the slide be slightly tilted. All these imperfections yield errors that depend on the position within the slide. PreP+07 allows to separate the slide in sectors and apply the preprocessing on each sector, making this procedures specific for each sector characteristics.

1.5.- Data acquisition

The devices used for measuring are neither perfect nor unlimited. Saturation and quantization appear when scanning, and can barely be removed. Saturation appears due to the finite nature of the devices that have a maximum response value. When a measure exceeds this maximum, the device can only read its maximum and usually with distortion. Quantization is due to finite word length in digital devices. Scanners are such devices. A technique for improving the quality of scanned data (García de la Nava, et al. 2004) is implemented in PreP. The corresponding visualization is also implemented and it is called 'intensity-intensity' (or I-I) plot. It shows the spots according to the intensity measured in a low-sensitivity scan and a high-sensitivity scan for both the green and red channels. This plot can also be used for comparison of two replicated slides or scans. If the replication is properly done, the spots must show a linear relation, otherwise, when the scans have different calibrations, the data will follow a non-linear curve due to saturation or calibration effects.

1.6.- Random errors

Random errors are a menagerie for all the imperfections, uncertainties and other factors that cannot be easily and safely corrected. The general approach to minimize this error is replication. Statistical methods are used for estimating the real value that these errors blur, but the results of such methods are not a definite and concrete answer. Only when the number of replications is large, the estimation is of confidence. There are several kinds of visualization that aids in the analysis of the replicated data. Dispersion, Deviation and Correlation of replicates are diagrams that expose these statistical values and their dependence on the average of the replicated spot intensities. All these graphs allow the quality estimation of the replication. For the dispersion graph, the data points should be along the diagonal, and the more noise, the more blurred they will appear. As a rule of thumb, the more spread the data have, the less quality. To assess the normality of replications, the QQ plot compares the quantiles of the normal distribution and the quantiles of the data distribution. If the data is nearly normal, this graph will be a diagonal line. One typical assumption is that the noise that perturb the data is normally distribute. This graph will test that hypothesis.

1.7.- Other visualizations

There are other visualization procedures not aimed at any specific error but which are complementary to the previous ones. These are the RG plot which has a similar use as the MA plot, the ratio density graph (also there is a per-block view) which is similar to the box graph and slide view with quality that shows the quality value of each spot in the blue channel. This last visualization is only available after some procedures that estimate the quality are

Table 1. Methods implemented in PreP+07 (*: new in PreP+07)

**Error Type**		**Causes**	**PreP+07 Procedure**
**Systematic**	Ratio shift.	Different dye or labelling efficiencies; different scanning sensitivities or laser power for different colours, non-linear transfer functions in photo detector.	Ratio correction via fitting curve, dye-swap.
	Contrast variations.	Nonlinearities and different conditions when measuring.	Scaling.
	Ratio shift.	Hybridization efficiency	Lowess and Supervised- Lowess*
	Spatial effects	Non-uniform solution spreading, irregular lighting, differences in print-tips; tilted slide.	Division of slide in sectors. (PrintTipLowess)
	Data acquisition (Saturation and quantization)	Finite dynamic range. Translating continuous values into discrete ones.	Double-Scan, Intensity thresholds and filtering.
**Random**	Multiple.	Imprecision due to parameters associated to the experiment.	Value estimation via replication.
	Generalized.	Intrinsic errors of the measure processes.	
**Statistics**	Error measures	Test used in inference which determines if the difference between a sample mean and the population mean is large enough to be statistically significant, that is, if it is unlikely to have occurred by chance.	Global and local z-test, t-test; etc.

2. AVAILABLE SOFTWARE PACKAGES FOR GENE-EXPRESSION DATA PRE-PROCESSING

Product	Company / Institute	OS	Preprocessing					License
			F	N	R	S	L	
Acuity: (http://www.axon.com)	Axon Instruments Inc.	W	Y	Y	Y		Y	C
AMADA/AMIADA (Xia and Xie, 2001) (http://dambe.bio.uottawa.ca/amiada.asp)	Dept. Biol. Univ. Ottawa	W		Y				F
ArrayVision (http://www5.gelifesciences.com/aptrix/upp01077.nsf/Content/microarrays_analysis_arrayvision)	GE Healthcare	W	Y	Y	Y	Y		C
BioConductor: (http://www.bioconductor.org)	Many	Many*	Y	Y	Y	Y	Y	FO(GPL)
BioMine (http://www.gnsbiotech.com)	Gene Network Science	W	Y	Y				CA
BRB array tools: (http://linus.nci.nih.gov/BRB-ArrayTools.html)	Mol. Stat. and Bioinf. Section, Biometric Res. Branch, NCI	W	Y	Y		Y	Y	FAN
DNA-Chip Analyzer (dChip) (http://www.dchip.org)	Wong Lab. Dept. of Stat., Hardvard Univ.	W	Y			Y		FA
Engene (García de la Nava *et al.* 2003) (http://www.engene.cnb.uam.es)	Comp. Archit. Dept., Univ. of Malaga.	Web	Y	Y				FR
Expression Profiler : http://ep.ebi.ac.uk	European Bioinf. Instit. (EBI)	Web						F
ExpressionSieve: http://www.biosieve.com	BioSieve	Java		Y				CA
GeneLinker, GeneLinker Platinum (http://microarray.genelinker.com)	Mol. Mining Corp.	W	Y	Y				CA
GeneMaths: http://www.applied-maths.com	Applied Maths	W		Y				C
GeneSight (Draghici *et al.* 2001) / Imagene: http://www.biodiscovery.com	BioDiscovery	WLM		Y			Y	C
GeneSpring: http://www.silicongenetics.com	Silicon Genetics	WLM		Y			Y	C
Genesis (Sturn *et al.* 2003) http://genome.tugraz.at	Bioinf. Group, Inst. Of Biomedical Eng, Graz Univ. of Technology	Java	Y	Y				F
GeneSense: http://www.inforsense.com	InforSense	?						C
GEPAS (Herrero *et al.* 2003) (http://gepas.bioinfo.cnio.es)	Bioinf. Unit, National Spanish Cancer Center (CNIO)	Web	Y	Y				F
J-Express: http://www.molmine.com	MolMine	Java	Y	Y			Y	C
MAExplorer (Lemkin *et al.* 2000) (http://maexplorer.sourceforge.net)	Open Source at SourceForge	Java		Y	Y			FO (MPL)
Microtracker: http://ocimumbio.com)	Ocimum Biosolutions	?	Y	Y				C
Partek Software Suites (http://www.partek.com) – Comercial	Partek	WLU		Y			Y	C
Soochika: http://www.strandgenomics.com	Strand Genomics	WLM	Y	Y				R
TIGR MIDAS (Saeed *et al.* 2003) (http://www.tigr.org)	The Inst. Of Genom. Res. (TIGR)	Java	Y	Y	Y		Y	FO
Xcluster: http://genome-www.Stanford.Edu/software	Standford Univ.	WLMU						FAN
X-Miner: http://www.x-mine.com	X-MINE	Web						C
Xpression NTI (http://www.informaxinc.com)	Informax Inc	W		Y				C

License: C: Commercial, F: Free, CA: Academic license available, FA: Free for academic, FAN: Free for academic and nonprofit, R: Upon request, O: Open source. Commercial products are shadowed.
Preprocessing: F: Filtering and Filling, N: Normalization (basic), R: Replication, S: Statistical Modelled Preprocessing, L: Nonparametric Preprocessing
Operating System: W: Windows, L: Linux, M: Mac, U: Unix
*: Bioconductor needs the R-Environment which has a textual interface and supports many OSes.

Table 2. Existing software specialized in gene expression data pre-processing

3. PROCESSING PROTOCOL

This section provides specific information about the protocol described to pre-process the datasets used in the experimental demonstration in this paper, including a representation of methods and graphs in PreP+07 (see figure 6).

3.1.- Steps in a typical analysis using PreP+07

For a complete data processing with PreP+07, the logical use of the available methods on it would be the next:

1. Column functionality selection in load screen (i.e. the flag column must be selected in case of doing a filter based on flags).
2. Data filtering, where many selections can be chosen on the basis of the spot quality, the presence/absence of genes, threshold fold change, etc.
3. 2scan resolution if scanning has been performed at different intensities.
4. Data quality checking, using normality graphs to confirm the normal distribution in order to apply the proper method in consecutive steps.
5. Data normalization to correct the deviation (lowess or supervised lowess).
6. Data scaling to avoid the comparison between probes.
7. Statistical significance calculation for each gene.

Technical details about some of the steps will be extended in the next sub-sections.

3.2.- Filtering Flagged spots

Genepix output from the scanner device provides an estimation of signal quality/confidence on a *Flag* labelled column. High confidence values set the flag to zero or un-flagged, and -50, -75 and -100 negative values are used for low quality spots with the following criteria:

"-50" values are assigned if a feature cannot be found during an auto align blocks.
"-75" values are assigned to missing values.
"-100" values are manually assigned by the researcher to spots that present hybridization problems such as external objects, washing problems, etc.

13056	Slide spots
896	Empties
12160	Spots
140	Negative control
12020	Spots with tomato material
6.053<br>5.967	Negative flag<br>Filtered

Table 3. Number and content of spots in the tomato exercise along the different filtering steps

3.3- Graphical representation of the initial quality of data for the slides used in the tomato experiment (complements Figure 4.)

Figures 7 and 8 display the initial state of slides before filtering and lowess steps and the results after the procedure (MA and box-plots graphs).

3.4.- Notes about replication

There are two kinds of replicates: biological and technical replicates. Biological replicates use RNA independently derived from distinct biological sources and provide both a measure of the natural biological variability and the system under study, as well as any random variation occurred in sample preparation; this is the kind or replicate that should contain a microarray design. Technical replicates include repeated spots in the array or replicated hybridizations with the same RNA; it only provides precision to data, but does not account for biological variation, so this kind of replicates must be reduced to the minimum.

3.5.- Details about using R

All the 3 slides belonging to condition B (in the tomato exercise) were dye-swapped regarding the condition A (control: green and target: red). For the appropriated management of data in R, it was necessary to modify –manually- the log-ratios sign in class B (dye-swap condition is used in R after the contrast test (this step was carried out with MeV program).

Additionally, in all steps that include a filtering option it was necessary to remove (manually again) those data with negative flag. Certainly, R does not take into account these spots to compute the fitting procedures, but could be marked as “significant” along the analysis (see figure 9) (we have checked that all points not-removed are considered for analysis with independence of the flag value).

4. RESULTS AND FIGURES FROM MUS MUSCULUS EXPERIMENT

With the aim of checking the similarity between the results produced using R (Limma package) and PreP+07, two different data analysis consisting of a pre-processing including Filtering and Lowess (FL) and other using Filtering, Lowess and Scaling has been performed.

After the analysis, the results were introduced in MeV program from TM4 package to calculate t-values for each gene in the experiment. These t-values were used to estimate the similarity in the predictions. In addition, results coming from FL analysis has been studied including a 0.05 rank of variation. The percentage of common genes in each rank is represented in figure 10.

For a better precision in the analysis, genes coming form PreP+07 analysis (FL protocol) with t-values less than 0.001 (0.005 produce 109 results) were studied individually and positioned in the list coming from R analysis (see table 4)

[1] ID	[2] P+07 pvalue	[3] P+07 rank	[4] R pvalue	[5] R rank	[6] pvalue differenc.
mMC022253	3.03E-04	1	0.003765	87	0.003462
mMR031140	4.54E-04	2	3.87E-04	2	0.000067
mMC010926	5.19E-04	3	5.05E-04	3	0.000014
mMR100301	5.28E-04	4	0.014242	441	0.013713
mMC019471	5.52E-04	5	6.07E-04	7	0.000055
mMC008837	5.74E-04	6	0.003398	73	0.002824
mMC002460	5.96E-04	7	5.72E-04	5	0.000024
mMR026341	6.04E-04	8	0.001496	24	0.000892
mMC023064	7.28E-04	9	6.59E-04	9	0.000069
mMC008038	9.90E-04	10	7.32E-04	10	0.000257

Table 4. Differentially expressed genes obtained with the FL protocol using PreP+07 contrasting their rank-position against Limma ranking. Columns correspond to: [1] gene ID; [2] and [4] t-test p-value for data processed by PreP (in increasing order) and Limma; [3] and [5] position in the list of significant genes in PreP+07 and Limma; [6] differences in p-value: | [2] – [4] |. T-test p-values were obtained using MeV from TM4 package.

The most differences in results appear in gene mMR100301, in fact, it would be the only gene not selected in a R analysis if we established the threshold in 0.01 or 0.05. For this reason it will be studied from the original data to check where were the changes produced (Table 5).

Gene	Sample	GSM319497	GSM319498	GSM319499	GSM319500	GSM319501	GSM319502	Mean A	Mean B	Dev A	Dev B
mMR100301	PreP+07 Log-ratio	0.1086	0.0915	0.0725	-0.2318	-0.2967	-0.2510	0.0909	-0.2598	0.0181	0.0333
	R LogRatio	0.1636	0.1752	0.1280	-0.1426	-0.2748	-0.1724	0.1556	-0.1966	0.0246	0.0694
	Differences	0.055	0.0837	0.0555	0.0892	0.0219	0.0786				

Table 5. Detailed view of mMR100301 gene including log-ratios (grey boxes), group means and deviations (infected versus not infected) obtained in both analysis (PreP+07 and R). Differences in log-ratios are also shown.

As the table shows, little differences produced in log-ratio values are the origin of important differences in means between R and PreP+07 and, what is more important, differences on their deviations. The higher differences in means between group A and group B produced in PreP+07, make this gene statistically significant, whereas means produced in R analysis are nearer and their deviations are higher. These two factors imply the selection of the gene in PreP+07 analysis and the rejection in the R analysis.

Excluding this particular case, if we set up a selection threshold at 0.005 or 0.01, all the genes selected using PreP+07 would match with the genes selected with R as can be seen at table X and figure X at these ranks, so the procedures integrated in both are completely equivalents, being easier the handling of PreP+07.

5. R USE ANALYSIS

We have included in http://www.bitlab-es.com/prep a large analysis of Bioconductor drawbacks made available in the ACGT European project (R analysis- context scenario - biostatisticians) in which our group is a partner. The structure of the document is the following, each section has three areas:

- **Context of Use:** resume about what is going to be discussed in that section.
- **Dialogue principle:** keywords about each context of use.
- **System requirements:** the conclusions about the lack of R software, the user needs.

A short summary of the most remarkable paragraphs are:

- R is missing some features, for example, interactivity, which is a critical feature. For instance, one cannot interactively visualize large clustering results (e.g., heat maps) with zooming capability, only static plots can be produced. Other interactive features are missing, such as menus. It would be nice to have a graphical environment where the user can use these functions with menus to click on a button to get the required information. A good visualization of the results of the analysis is compulsory.

- It would be important to provide a mechanism to “replay” the user actions such that a specific graphics can be reproduced (e.g. addition of legends, change of axes, lines and points styles, change of viewpoint in 3D plots, etc…).

- A major drawback in the use of R is the inconsistent specification of the defaults for the functions between versions of the software (or, for a given version, between different platforms supporting the code). For instance, while the default character to specify comments to read in text files was the hash sign in some early versions of the code, this was changed to have no default comment sign.

Regarding software usage, factors of stress are:

- Strong differences in user interface between versions of the code.
- Differences in code outputs for the same input when different versions of the code are used (e.g. if default parameters of an algorithm are changed).
- Lack of clear documentation (written in simple and correct English), full documentation in a correct and simple English is required.
- Unclear error messages, error messages have to be clear and understandable for the user.
- Arbitrary change in code output, depending on unrelated changes in the input (e.g. in R, matrices converted to vectors depending on dimensions).
- Lack of ways to report bugs.
- Lack of ways to have bugs corrected, the user should have the possibility to report bugs and get the correction effectively.
- Lack of ways to have support (either from the community of users or from the developers)

6. TECHNICAL DETAILS OF PREP+07 CODE

Objects and Classes

- Project: this is the main object that stores the information about the performed operations. The project is composed by a lis t of states.
- State: This object represents a slide that is the result of a step. The first state is the result of the load step.
- *Slide*: Initially, the list of spots to be read by the data analysis program. It will be also necessary to add information about the slide structure by blocks or sectors, the operations made over the slide, the replications groups, etc.
- *fit*: a fit is done over a set of spots of a slide,
- *Spot*: intensity of the different channels, this information is derived as the logarithms of those values. A spot also has some tags that contain extra information about it but that won't be used in the processing methods.

Relations

- A project contains one or more states.
- A state contains one or more slides.
- A slide contains zero or more fits.
- A slide contains zero or more replication groups.
- A slide contains one or more spots.
- A spot contains zero or more tags.

Operations

- Over a project:
  - Load of the microarray file.
  - Load and save of the project.
  - Selection of the visualization method.
- Over each slide:
  - Block division
  - Lowess/Supervised fit
  - Replication specification
  - Scaling
  - Double Scan method
  - Statistical test
  - etc..
- Over the last step
  - Apply an adjust
  - Data saving

7. BASIC CLASSES DIAGRAM USED IN PREP+07

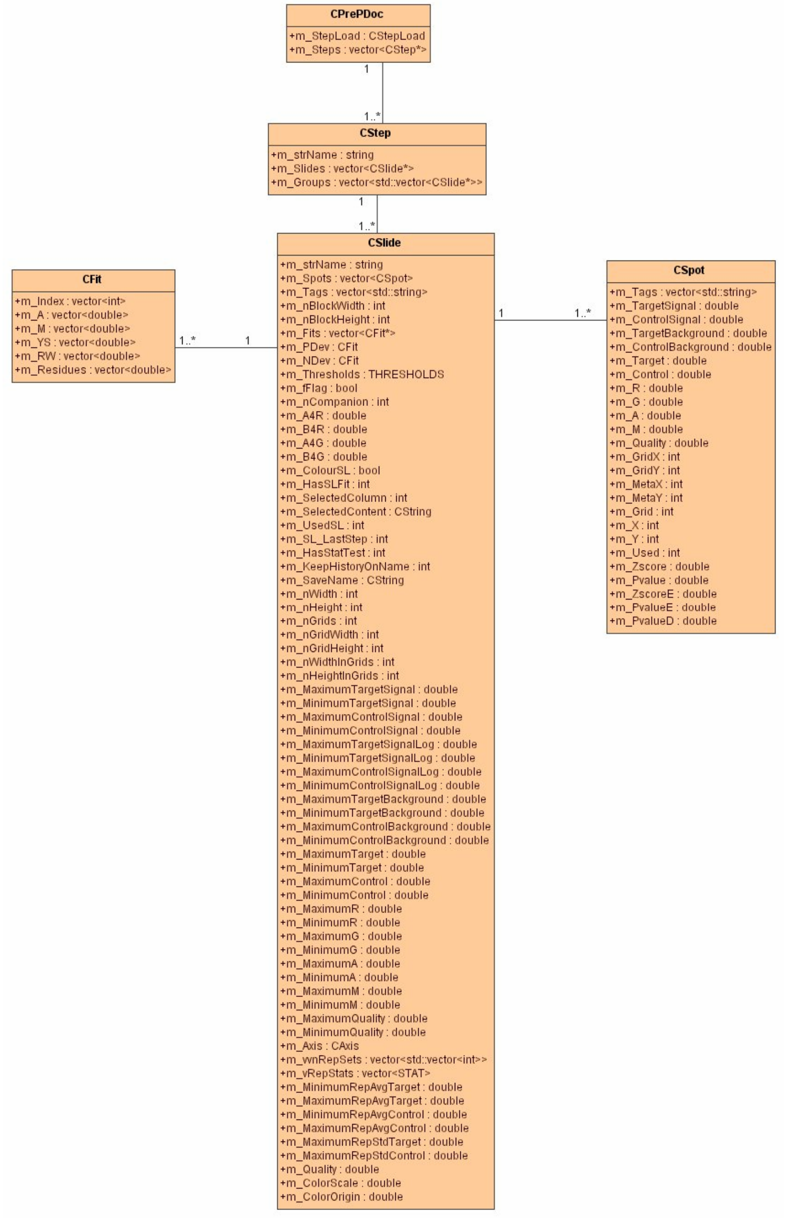

8. MANUSCRIPT'S IMAGES IN FULL RESOLUTION

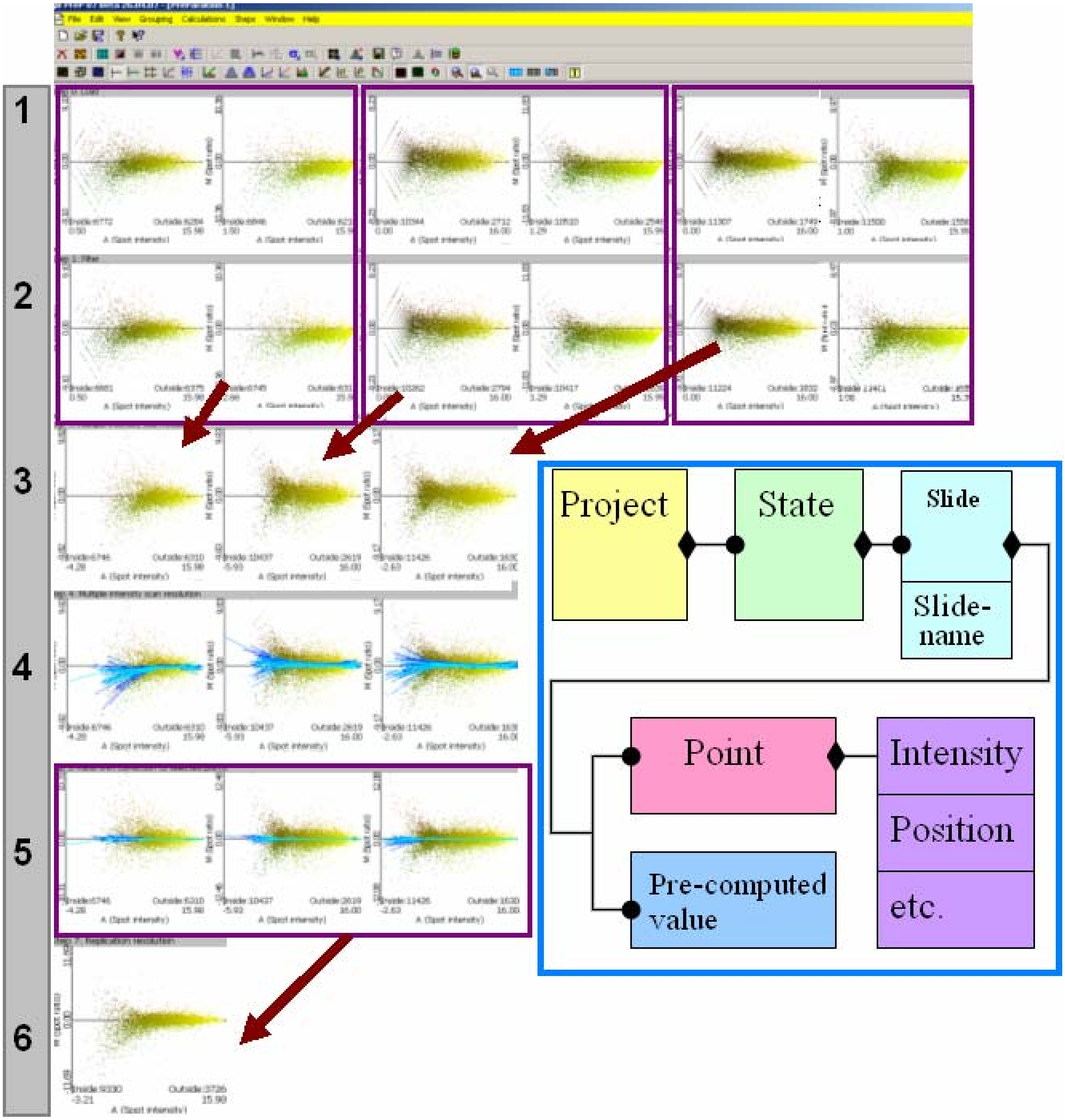

Figure 1. Typical steps in a complete analysis of gene expression (by row): (1) Filtering empty spots; (2) double scan resolution; (3) Lowess estimation of parameters;, (4) applying the Lowess estimation; and (5) Replicates resolution. Inside the box: object diagram of a Pre*P+07* project, where diamonds represent “is composed of” and circles represent “one or more”

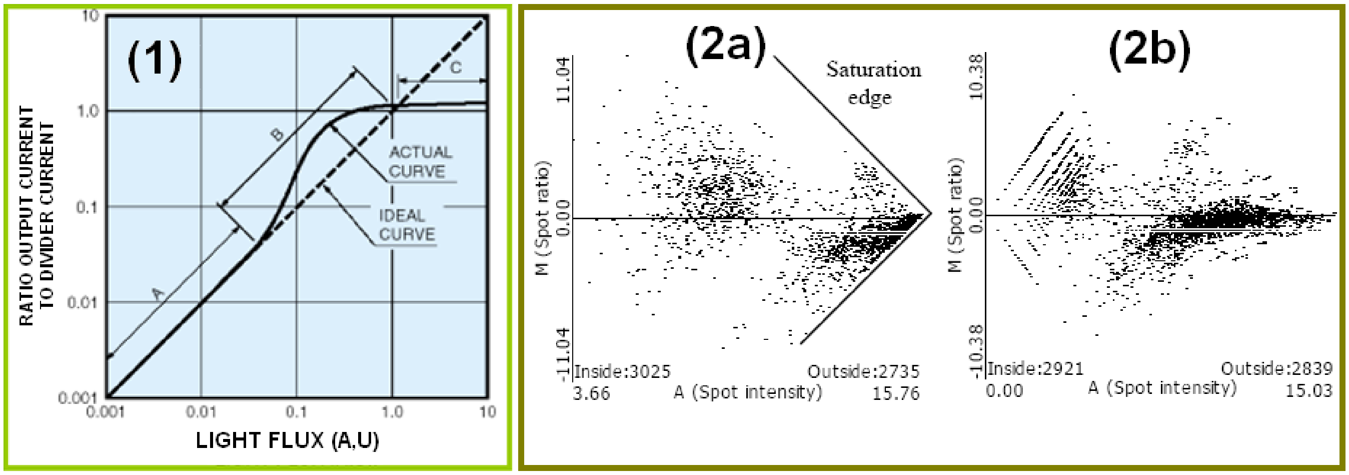

Figure 2. Double-Scan procedure: In (1) the transfer function of the photo-detectors used in the scanners is depicted. At high intensities the relationship between the incident light level and the output current begins to deviate from the ideal intensity in an effect called saturation that typically drawn an arrow-shape (see 2a). On the other hand, quantization occurs when digitizing. All the unlimited physical values have to be encoded by a reduced set of discrete values, producing the same value for a range of different values (see Gray and Neuhoff, 1998). This effect can be observed in (2b) as a set of parallel lines. 2-Scan strategy (Garcia de la Nava, et al. 2004) is based on the rather simple idea of producing two images with different calibrations from which a mathematical model produce a coherent but extended range of values.

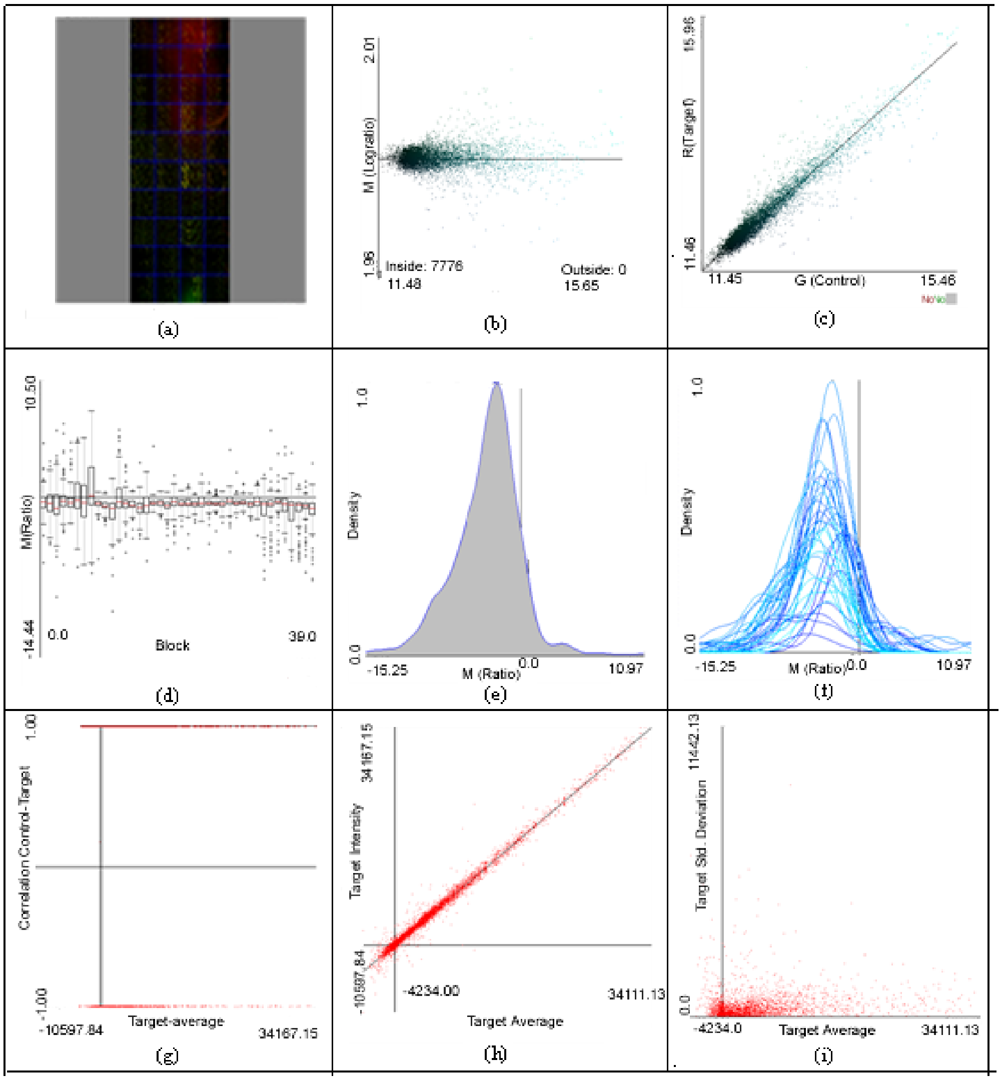

Figure 3. Some PreP+07 views. a) lide view, b) slide view of coherent data, c) slide view with quality, d) MA plot, e) RG plot, f) box graph, g) density distribution of ratios, h) density distribution of ratios within each sector, i) intensity-intensity plot, j) replicated spots vs. their average, k) deviation of replicated spots vs. their average, l) correlation of replicated spots vs. their average, m) normality of replications, a QQ graph for normality check, n) MA plot with thresholds for filtering, o) MA plot with Lowess fit and p) intensity-intensity plot with regression curve and threshold.

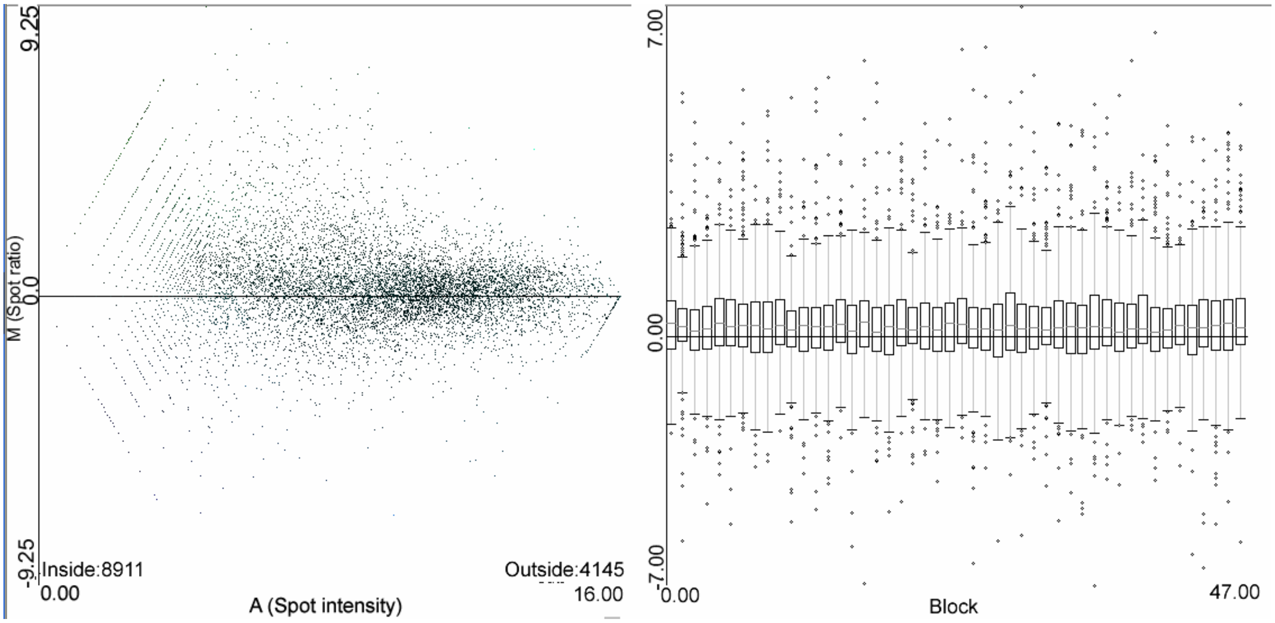

Fig.ure 4. MA and boxplot graphs on one of the initial dataset in tomato experiment. Quantized low-quality values can be observed in the low intensity zone of MA graph suggesting the need for filtering procedure, and the nice shape in the box-plot (on the right hand side) suggest that scaling procedure is unnecessary.

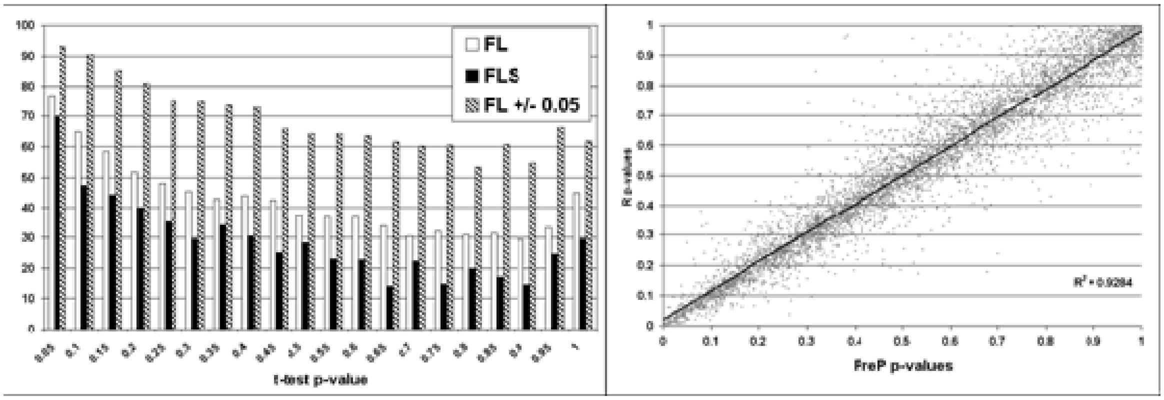

Figure 5.- Percentage of predicted genes by Limma in the same p-value range of PreP+07 predictions at Solanum Lycopersicum experiment. White bars belongs to protocol 1 (FL), black bars correspond to protocol 2 (FLS) and slashed bars belongs to protocol FL with neighboring (a range of ±0.05). Note the high coverage value (>90%) for the most significant genes (p-value < 0.1), and major differences are produced in the low quality expresión levels. The general coverage is approximately 70%.

9. SUPPLEMENTARY MATERIAL’S FIGURES

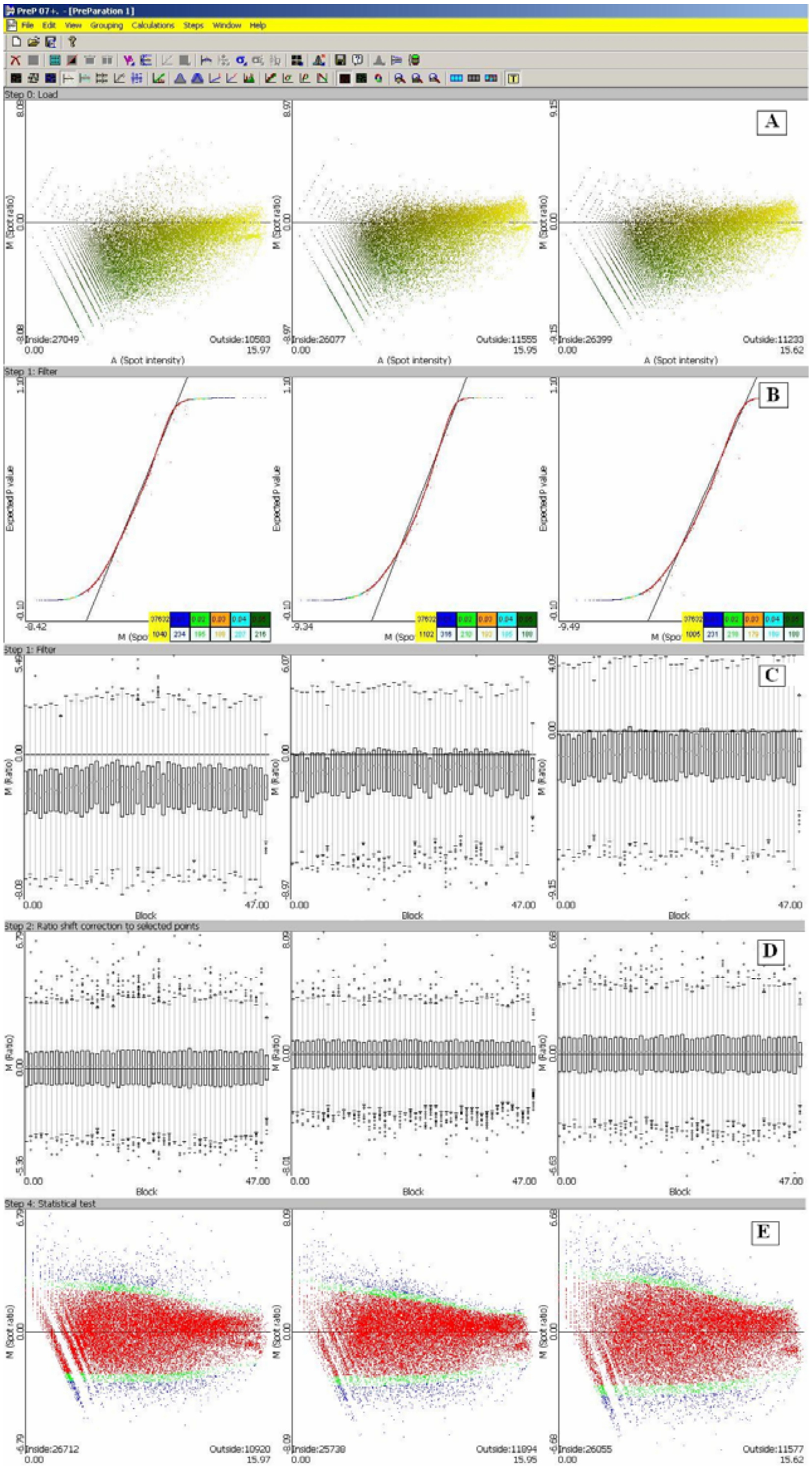

Figure 6. Different states when applied a typical protocol for pre-processing using PreP+07 (each row represents a state): **[A]** Initial state of loaded chips belonging to mock-infected mouse chips (GSM319497 to GSM319499) in MA representation. A clear ratio shifting can be observed. **[B]**Data normality evaluation using PN graphs. When the lines are nearer to the diagonal, normal the dataset is. **[C]** Box-plot data visualization before lowess adjust. Again, data ratio shifting can be noticed, in the other hand no high differences in scale appear between slides. **[D]** Box-plot visualization of slides after lowess adjust. **[E]** MA quality graph where significant genes are in blue. Genes in green are in a less confidence interval.

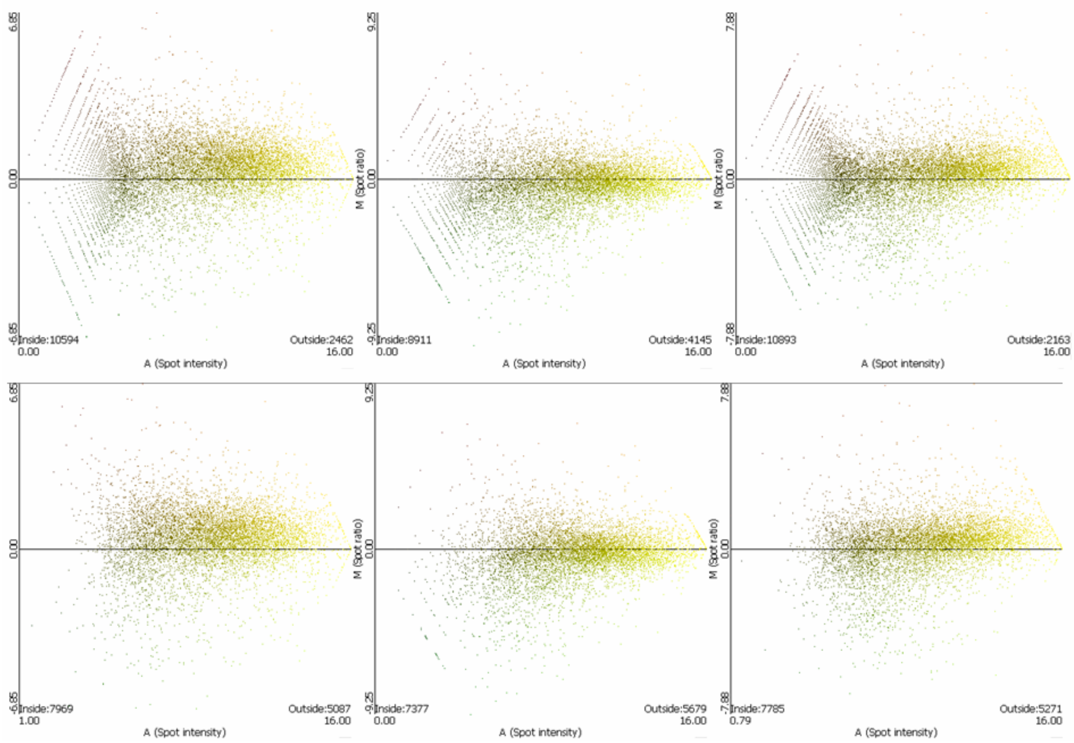

Figure 7. MA graph representing class A from tomato experiment in conditions of pre-filtering and post-filtering. Most of genes at low intensity presenting quantization have been removed.

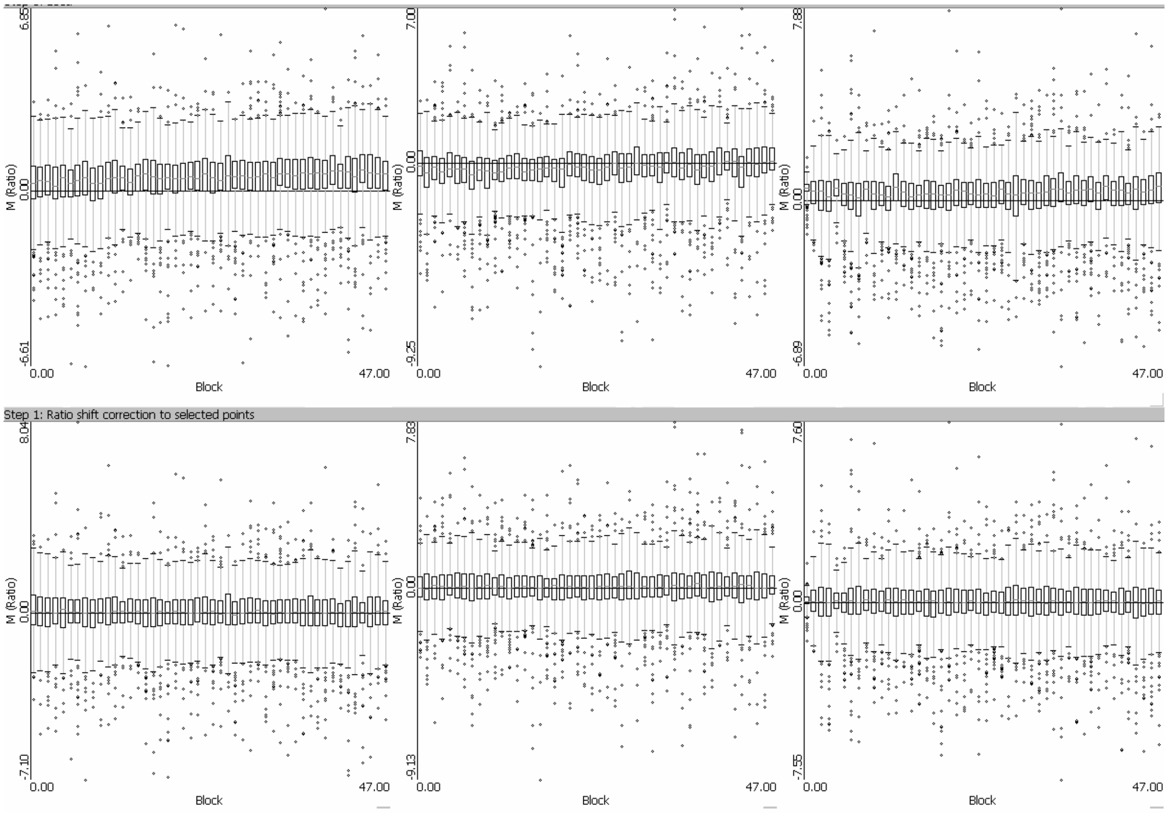

Figure 8. Box-plot visualization of class A in tomato experiment before and after lowess adjust.

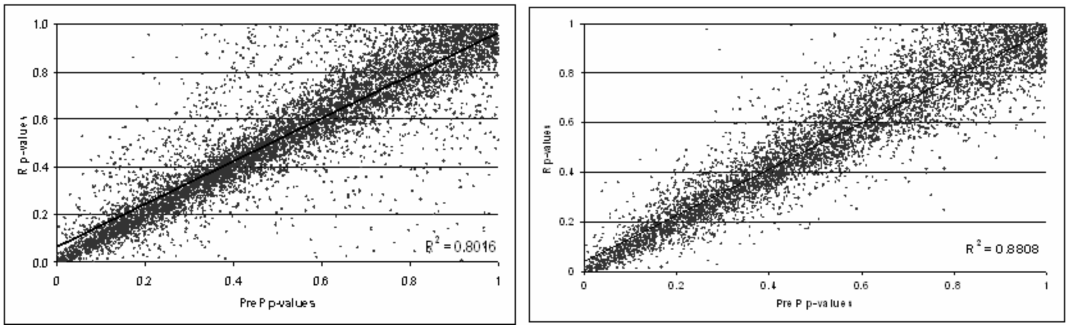

Figure 9. Correlation between p-values obtained using R and PreP+07. Picture on the left shows all spots, and in the right when flagged spots are filtered.

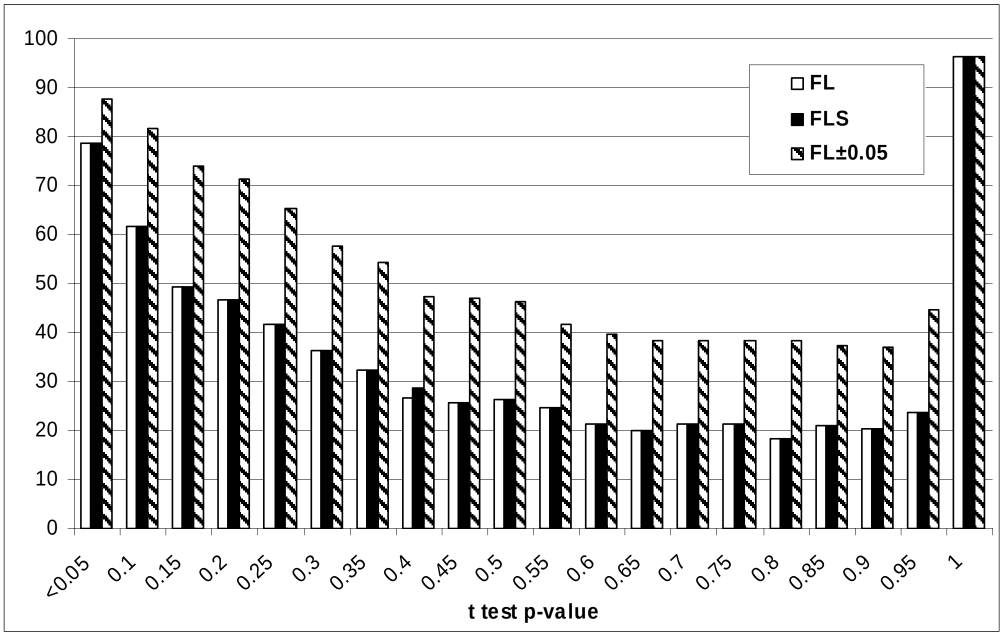

Figure 10.- Percentage of predicted genes by Limma in the same p-value range of PreP+07 predictions at Mus musculus experiment. White bars belongs to protocol 1 (FL), black bars correspond to protocol 2 (FLS) and slashed bars belongs to protocol FL with neighboring (a range of ±0.05).
